# Dermatological remedies in the traditional pharmacopoeia of Vulture-Alto Bradano, inland southern Italy

**DOI:** 10.1186/1746-4269-4-5

**Published:** 2008-02-06

**Authors:** Cassandra L Quave, Andrea Pieroni, Bradley C Bennett

**Affiliations:** 1 Department of Biological Sciences and Center for Ethnobiology and Natural Products, Florida International University, 11200 SW 8^th ^St., HLS 320, Miami, FL 33199, USA; 2 Division of Pharmacy Practice, School of Life Sciences, University of Bradford, Richmond Road, Bradford BD7 1DP, UK

## Abstract

**Background:**

Dermatological remedies make up at least one-third of the traditional pharmacopoeia in southern Italy. The identification of folk remedies for the skin is important both for the preservation of traditional medical knowledge and in the search for novel antimicrobial agents in the treatment of skin and soft tissue infection (SSTI). Our goal is to document traditional remedies from botanical, animal, mineral and industrial sources for the topical treatment of skin ailments. In addition to SSTI remedies for humans, we also discuss certain ethnoveterinary applications.

**Methods:**

Field research was conducted in ten communities in the Vulture-Alto Bradano area of the Basilicata province, southern Italy. We randomly sampled 112 interviewees, stratified by age and gender. After obtaining prior informed consent, we collected data through semi-structured interviews, participant-observation, and small focus groups techniques. Voucher specimens of all cited botanic species were deposited at FTG and HLUC herbaria located in the US and Italy.

**Results:**

We report the preparation and topical application of 116 remedies derived from 38 plant species. Remedies are used to treat laceration, burn wound, wart, inflammation, rash, dental abscess, furuncle, dermatitis, and other conditions. The pharmacopoeia also includes 49 animal remedies derived from sources such as pigs, slugs, and humans. Ethnoveterinary medicine, which incorporates both animal and plant derived remedies, is addressed. We also examine the recent decline in knowledge regarding the dermatological pharmacopoeia.

**Conclusion:**

The traditional dermatological pharmacopoeia of Vulture-Alto Bradano is based on a dynamic folk medical construct of natural and spiritual illness and healing. Remedies are used to treat more than 45 skin and soft tissue conditions of both humans and animals. Of the total 165 remedies reported, 110 have never before been published in the mainland southern Italian ethnomedical literature.

## Background

The folk-medical treatment of dermatological conditions is prevalent in southern Italy and elsewhere. Dermatological conditions are particularly common in rural agro-pastoral communities, where skin abrasions and small cuts are regularly exposed to bacteria found in the soil and animal faeces. At least one-third of all traditional remedies used in the south Italian folk pharmacopoeia are directly relevant to the skin.

Skin and soft tissue infection (SSTI) caused by multidrug-resistant bacteria, such as methicillin-resistant *Staphylococcus aureus *(MRSA), are an ever-increasing source of death and high healthcare costs worldwide. In the US alone, over 126,000 hospitalizations each year are due MRSA infections [1]. Moreover, invasive MRSA infections affect an estimated 94,360 individuals each year in the US and are associated with approximately 18,650 deaths, exceeding mortality estimates for AIDs [2]. Natural products from botanical sources used in traditional folk remedies for the skin may offer a new route for combating multidrug-resistant bacterial infections through the elucidation of biological compounds with novel mechanisms of action. Furthermore, validation of traditional remedies could rekindle community interest in preserving local traditional medical knowledge.

Ten communities in the Vulture-Alto Bradano area of the Basilicata (historically known as Lucania) province were the focus of this study. Basilicata, a region of southern Italy bordering the Tyrrhenian and Ionian Seas, is divided into two Provinces: Potenza and Matera. It is bounded by the regions of Puglia (north and east), Calabria (south), and Campania (west). The territory is roughly divided into a mountainous western section, which is dominated by the Appennino Lucano, an eastern section of wide valleys and low hills, and flat plains to the south along the Ionian Sea.

Basilicata region covers 9,992 km^2^, and based on a 2001 census of The Italian National Statistical Institute (ISTAT), Basilicata has a population of about 600,000. ISTAT also reports that the Basilicata region has the lowest percentage of urban population (17%, calculated in the period of 1997–1999), the highest life expectancy (75.7 years, calculated in the period 1991–1995), and presents the lowest utilization of allopathic medical services (23.9% among men, 32.5% among women, calculated in 1997) in all of Italy [3].

Basilicata has been influenced by historical immigration flux from both Greece and Albania. Today, immigrants come mainly from Eastern Europe or from Northern Africa. The majority of recent immigrants into the smaller communities, such as those included in this study, come from Eastern Europe seeking work as housekeepers and caretakers for the elderly, whereas North Africans typically seek employment in the larger cities.

The Vulture-Alto Bradano area of northern Basilicata is characterized by its proximity to the dormant volcano, Monte Vulture (1,326 m.a.s.l.). The soil of this region is particularly rich due to the presence of tuffaceous-clayey-volcanic soil (produced by the explosive volcano ca. 830,000 years ago) and the local economy is founded in large part on agriculture. Durum wheat, olives, and the *aglianico *variety of grapes are the predominant crops. Small-scale pastoral activity is also important, and goat and sheep cheeses are produced locally. Regional industry, other than agriculture, is scarce, but some local artisans produce ceramics and a substantial portion of the population between 20–45 years old are currently employed in the Fiat automobile factory or other associated factories.

Based on data from historic ethnic Albanian communities in the Vulture-Alto Bradano, traditional knowledge (TK) related to household remedies corresponds with age and gender. The likelihood of TK transmission is affected by work patterns, which influences the amount of time an individual spends in the natural environment performing agricultural work. Women are the primary carriers of knowledge for folk remedies, as well as the primary providers of household medicine. Older generations are more knowledgeable of plant sources of both wild foods and medicines [4-8]. Furthermore, because of the strong link between TK and age, the level of TK in small communities is on a steep decline as the oldest generation dies. This reflects a sense of urgency to record folk remedies, which are normally passed down through oral tradition, before they are lost forever.

In the past 50 years, only a few ethnobotanical studies have been focused on Basilicata [4-14]. The aim of this study is to collect ethnomedical data regarding the application of traditional remedies from botanical, animal, and mineral sources for the topical treatment of the skin. In addition to remedies for humans, we also discuss certain ethnoveterinary remedies that are particularly important for the care of livestock in the study area. Socioeconomic dynamics are discussed in relation to the transmission of knowledge for folk remedies across generations. Botanical species identified as components to traditional remedies for SSTIs in this study will be evaluated for their potential in modifying MRSA growth and virulence in subsequent studies.

## Methods

**Figure 1:**
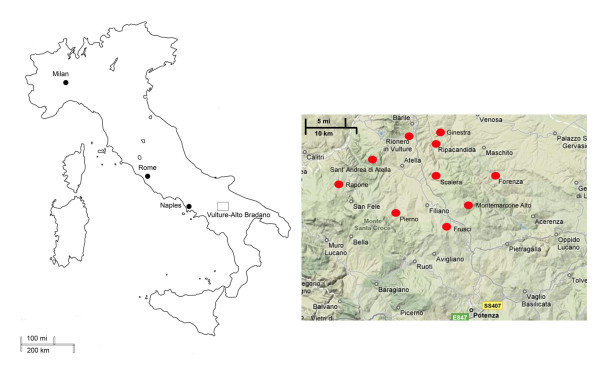
Map of the study region: the Vulture-Alto Bradano region of Basilicata, southern Italy.

Field research was conducted in ten communities situated in the Vulture-Alto Bradano area of Basilicata, Italy from April-July of 2006 (Figure 1). Random sampling techniques were employed to recruit 112 interview subjects. Interviewees were equally stratified by four age groups (21–35, 36–50, 51–70, 71+ years) and gender in order to compare TK levels across different generations. Prior informed consent, as approved by the Florida International University Institutional Review Board (#120505-01), was obtained before conducting interviews. We followed the ethical guidelines adopted by the ICE/International Society of Ethnobiology [15] and Italian Association of Ethno-Anthropologists (AISEA) [16]. We conducted interviews in Italian, took notes, and made audio or video recordings of the interviewees when possible. Data was collected using semi-structured interviews, participant observation, and focus groups. Interviewees were questioned about the medicinal uses of the local flora and fauna, particularly those related to the skin. Demographic data regarding each community sampled in the study is reported in Table 1.

**Table 1:** Data on the sampled communities [45–47]

***Community***	***Elevation (m.a.s.l.)***	***Number of Families***	***Number of Inhabitants***	***Number of Interviewees***	***% of Population Sampled***
Forenza	836	1,051	2,546	15	0.6%
Frusci (locality of Avigliano)	925	70	192	8	4.2%
Ginestra	675	280	726	10	1.4%
Montemarcone Alto (locality of Avigliano)	900	15	46	5	10.8%
Pierno (locality of San Fele)	960	24	52	5	9.6%
Rapone	838	473	1,203	9	0.7%
Rionero in Vulture	647	4,738	13,423	4	0.03%
Ripacandida	622	744	1,767	14	0.8%
Sant'Andrea (locality of Atella)	716	55	176	27	15.3%
Scalera (locality of Filiano)	670	185	488	15	3.07%

**Total**	**-**	**7,635**	**20619**	**112**	**0.54%**

All plants mentioned by interviewees were collected and identified following the standard work of the Italian flora [17]. Familial nomenclature follows the current Angiosperm Phylogeny Group [18]. Voucher specimens were deposited at the *Università degli Studi della Basilicata *(HLUC) in Potenza, Italy and Fairchild Tropical Botanic Gardens (FTG) in Miami, FL, USA. Bulk samples (100–300 g per species) were collected for bioassays. Plant materials were shipped to the USA under USDA permit #DP63438.

The transcription of vernacular names of traditional remedies described in the local pharmacopoeia follows the rules of the Italian language. The neutral centralized vowel 'schwa' of the southern Italian (Calabro-Neapolitanean) dialect spoken in the studied area, often marked by some linguists as (') has been denoted in this study with the symbol "ē". The collected data have been compared with all of the ethnobotanical studies conducted thus far in mainland southern Italy, including Abruzzo, Molise, Campania, Puglia, Calabria and Basilicata.

Statistical analyses of data, including calculation of mean values and standard deviations, were calculated using Microsoft Excel. Differences between means were analyzed with a one way ANOVA, followed by a series of 2-sample t-tests on SPLUS software. Differences were considered significant with P-values < 0.05.

## Results and Discussion

### The folk medical construct

Skin and soft tissue infections (SSTIs) are treated in different manners, depending on the perceived causation of the illness. Illnesses with a naturalistic (biological) causation are treated in a non-ritual context and involve the topical application of either a plant, animal, mineral, or industrial product as the therapeutic agent. This is usually carried out by the individual being treated or by the female head of household (mother or grandmother).

Illnesses of perceived spiritualistic causation are treated much differently. Some manifestations of inflammation on the skin, for example, are believed to be caused by malevolent spirits, such as *mal vjntē *(wind illness) or *fuoco morto *(dead fire illness). These cases are treated ritualistically, and a plant, animal, mineral, or industrial product is employed strictly as a ritual object in the ceremony, rather than as the therapeutic agent. Furthermore, the ritual treatment can be performed only by specific healers (most of whom are women) who are recognized in the community for their healing powers. The treatments for south Italian spiritual illnesses in Basilicata have been discussed in previous publications [19-21].

### Botanical remedies

We documented the topical application of 38 plant species from 27 families, comprising 116 distinct remedies (Additional File 1). Plants are listed alphabetically by species. The vernacular and English names are also listed, as well as the author citation of each species. Detailed information regarding the cultivation status, part(s) used, preparation, application and popular use is provided. A consensus index is included and is specific to each individual remedy cited. References to similar remedies in the ethnomedical literature on mainland southern Italy are noted.

**Figure 2:**
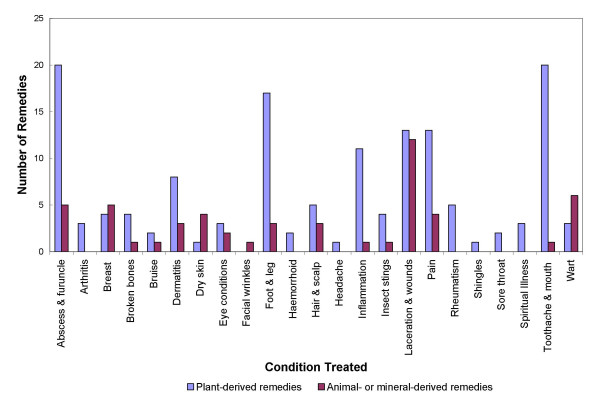
Number of plant- or animal- and mineral-derived remedies for the topical treatment of medical conditions in humans.

Some of the more common uses of these remedies include analgesic, vulnerary (especially for burn wounds), toothache, anti-inflammatory, anti-sting/anti-itch (for insect bites), antiseptic (for lacerations), anti-furuncle, haemostatic, suppurative, emollient, and anti-abscess. Roughly 14% of plant-based remedies are applied for the treatment of abscess and furuncle (Figure 2). Here we discuss three of the most frequently cited plant remedies: common mallow, white horehound and German chamomile.

#### *Malva sylvestris* (Malvaceae) – *malva*

Common mallow is a herbaceous species common throughout most of Europe, except in the most northern regions. It is one of the most important medicinal species in the southern Italian folk pharmacopoeia. Its use as a panacea is reinforced by a local saying, *La malva, da ogni mal' ti salva*, (the common mallow saves you from every disease). Common mallow is most often cited for its restorative properties for cold, flu, and stomach-ache and as a post-partum depurative [6,11]. In these cases, a decoction of the aerial parts is drunk.

It is also important in topical remedies for the treatment of toothache due to dental abscess or decay, heat- and diaper- rash, bruise, furuncle, abscess, and mastitis. The application of remedies from this plant to a number of SSTIs, most of which are typically associated with bacterial infection, suggests that this plant may exhibit antibacterial properties and should be subjected to further study.

#### *Marrubium vulgare* (Lamiaceae) – *maruggē*

White horehound is a perennial herb native to Europe, northern Africa, and temperate Asia. Much like common mallow, white horehound is also an extremely important species in the folk pharmacopoeia of southern Italy. It, too, is considered a panacea and is associated with the following saying, *A maruggē, ognē malē struggē *(the white horehound destroys every disease). In previous studies on south Italian ethnopharmacology, the use of white horehound decoctions as an expectorant, hepatoprotective agent, and cure-all has been described [6,7,11]. The Commission E monographs approve of white horehound use for treating loss of appetite and dyspepsia [22].

A decoction of the aerial parts can be used as a rinse to treat several important SSTIs, including general dermatitis, athlete's foot, furuncle, abscess, cyst, and wart in both humans and animals. Investigation of the anti-MRSA potential of this species and its phytochemical components could be promising.

#### *Matricaria recutita* (Asteraceae) – *camomilla*

**Figure 3:**
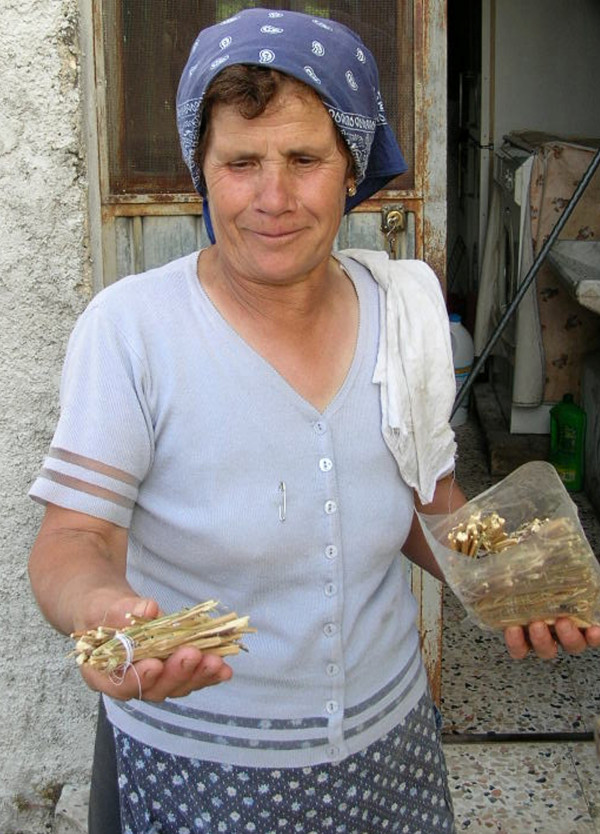
Mecca Maria Leonarda (63 yrs. old) uses decoctions of dried chamomile (*Matricaria recutita*) stems to treat eye infections.

German chamomile is best known for its anxiolytic and sedative properties. In the Vulture-Alto Bradano, it is also commonly used as a wash for skin conditions such as rash, acne and dermatitis and also as an anti-conjunctivital eye-wash (Figure 3). Application of German chamomile decoctions for the treatment of eye inflammation and infection has been broadly reported in Italy. German chamomile has demonstrated *in vitro *anti-staphylococcal activity [23] and has been found to promote wound healing *in vivo *[24]. The German Commission E has approved the use of German chamomile in external applications for the treatment of skin and mucous membrane inflammation, bacterial skin disease, and ano-genital inflammation [22].

### Zootherapy and remedies of mineral or industrial origin

The treatment of ailments with remedies made from animals and their products is known as zootherapy [25]. Since ancient times, zootherapy has been integral to the traditional pharmacopoeias of many cultures [26-28]. Despite its prevalence in traditional medical practices worldwide, the phenomenon of zootherapy has often been neglected in comparison to the literature regarding medicinal plant therapies. Animal-based remedies represent an important portion of traditional medicine practiced today in southern Italy. The sources of these remedies include human, pig, bee, chicken, and slug mucous.

Remedies of mineral and industrial origin are also important, and include sources such as copper sulphate, salt, soot, and old gas, among others. They are employed in the treatment of burn wound, furuncle, facial wrinkle, abscess, broken bone, bruise, chapped skin, conjunctivitis, bronchitis, wart, acne, stomach ulcer, callus, cradle-cap, and dermatitis.

Forty-nine remedies from 30 animal, mineral, or industrial sources are used in topical applications (Additional File 2). Remedy sources are listed alphabetically by English name. Vernacular names are also listed, along with detailed information regarding the preparation, application and popular use of the remedy. A consensus index is included and is specific to each remedy cited. References to similar remedies in the ethnomedical literature on mainland southern Italy are noted. Here, we discuss some of the most unusual of these remedies, including aged pig fat, garden slug and human breast milk.

#### Aged pig fat

Aged pig fat, locally known as *sugna fracidē*, is prepared by aging the dorsal back fat of a pig in a cool dark place, such as a *cantina*. It is an important component of several remedies for skin conditions. In addition to its emollient properties, it is also reported to be a useful vulnerary agent in the treatment of both animals and humans. It is also applied together with the leaves of *Rubus ulmifolius *to treat furuncle, and to draw the pus out of purulent abscess. A similar therapy with *R. ulmifolius *was reported in Campania, another area of southern Italy [29].

#### Garden slug

**Figure 4:**
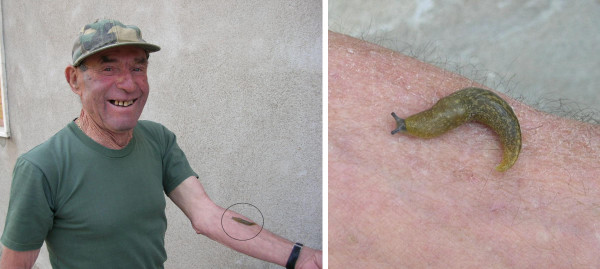
Vito Sabato (74 yrs. old) uses mucous produced by the garden slug (*Arion hortensis*) to treat warts, inflammations, and calluses on the skin.

Therapies involving the slug *Arion hortensis*, locally known as *u' marruculē *or *lummachē senza guscio *(snail without a shell), has been briefly mentioned in previous work by our group [11]. The most popular use of this gastropod is to treat gastritis or stomach ulcer by swallowing it whole and alive. A clear mucous produced by the slug is rubbed onto the skin to treat dermatitis, inflammations, calluses, and acne (Figure 4). The mucous is thought to promote wound healing. In addition, a special ritual is incorporated in the treatment of wart. Mucous from a live slug is first rubbed onto the wart, and then the slug is hung out in the sunshine to dry out and die. It is believed that once the slug has dried up, the wart should as well. A similar remedy for wart involving mucous from an unidentified snail is mentioned in Guarrera's work in Latium, Central Italy [30]. This may, however, be a reference to the same species of garden slug.

#### Human breast milk

Human breast milk is a popular zootherapeutic remedy, and is applied in the treatment of conjunctivitis, chapped nipples, and cradle-cap. The topical application of breast milk to infected eyes was cited by nearly one-third of the interviewees. This remedy was also frequently cited during a previous field study by our group in the Lucanian Dolomite region of Basilicata [11]. This use of breast milk may be well-founded due to the antibacterial properties of xanthine oxidase and IgA antibodies found in the colostrum and milk [31,32].

### Ethnoveterinary remedies

Ethnoveterinary practices have been reported on in Italy in the regions of Basilicata [13], Calabria [33], Tuscany [34], Romagna [35], Marche, Abruzzo, and Latium [36,37]. Much of this data on Italian ethnoveterinary medicine has been summarized in a useful database [38].

**Figure 5:**
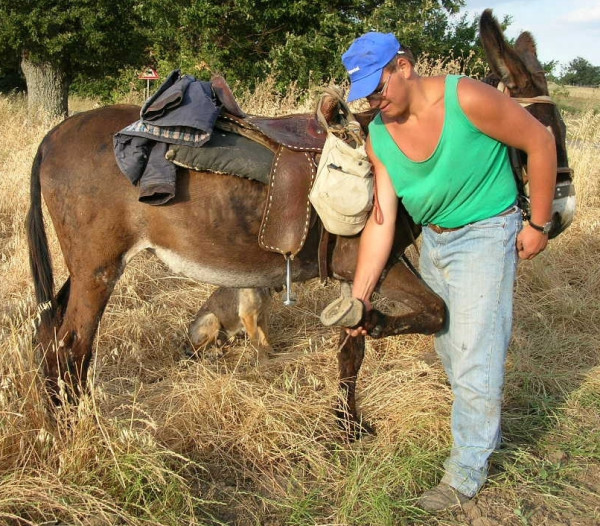
A young shepherd (23 years old) from Sant' Andrea uses a mixture of red wine vinegar and copper sulphate to rinse the cracked hooves of his ass. He also applies the crushed mature fruits of *Sambucus ebulus *(*Iervolē*) to open wounds on his livestock as an insect repellent.

In Vulture-Alto Bradano, the topical application of traditional remedies to treat wounds and inflammation in livestock was particularly important in the smallest communities surveyed, primarily in the localities of Montemarcone Alto, Sant'Andrea, and Pierno. Many families in these communities are economically dependent on agropastoral activities, and the health of their animals is a central concern (Figure 5). Plant, animal, and mineral (or industrial) derived remedies for ethnoveterinary applications are indicated with the symbol '♣' in Additional Files 1 and 2. Here, we briefly discuss two important sources of ethnoveterinary treatments: copper sulphate and St. John's Wort.

#### Copper sulphate

**Figure 6:**
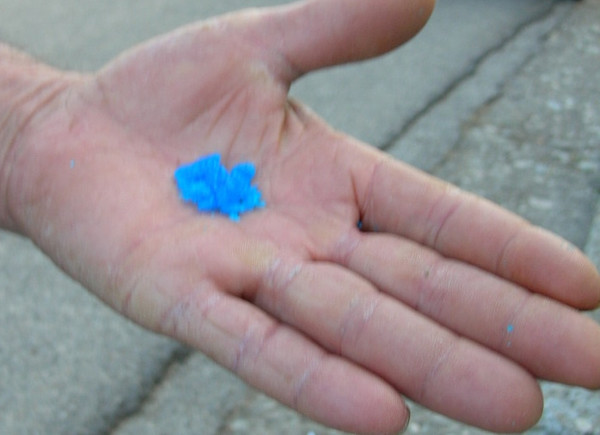
*Verderammē *(copper sulphate) is used in ethnoveterinary remedies for injured or infected hooves of livestock.

Copper (II) sulphate (*verderammē*) is a bright blue-green mineral sold locally as a chemical fungicide for plants (Figure 6). However, it is also one of the most frequently cited traditional ethnoveterinary remedies. While it is also used for the treatment of swollen feet in humans on occasion, the burning sensation it produces is a significant detractor. It is prepared either as a ground powder, a mixture with vinegar, or a mixture with water and salt and is then applied to cracked hooves or to the chapped skin surrounding the hooves of livestock. This ethnoveterinary remedy has also been reported in the western Basilicata Dolomite mountain range [10].

#### *Hypericum perforatum* (Hypericaceae) – *iperico*

An oleolite made from an olive oil infusion of the aerial parts of St. John's Wort (known locally as *erva pē rē cuttē *or *iperico*) is massaged into the legs of horses and asses to treat tendonitis. This remedy has also been described in Tuscan ethnoveterinary practices [34]. In other studies, oleolite preparations of the aerial parts or flowering tops have been reported for wound healing and to restore skin damaged by burns [36,39,40] or for treating mastitis in milk-producing goats [29]. Oleolite preparations of a similar species, *H. perfoliatum*, have been reported in Sicily for use as a disinfectant and to promote healing of burn wounds [41]. The anti-staphylococcal properties of *Hypericum *species are well known [42-44] and are likely relevant to these traditional therapies.

### Traditional knowledge: current trends and future projections

**Figure 7:**
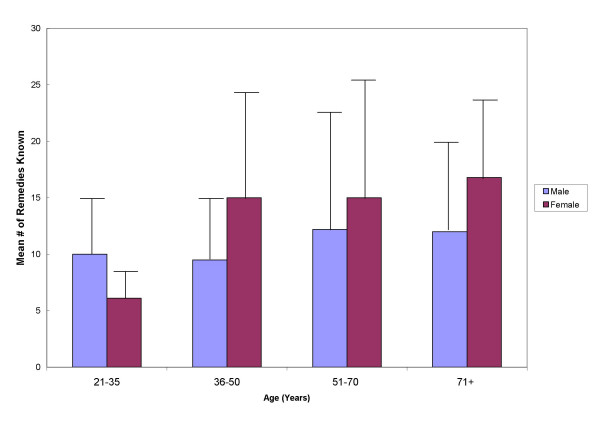
Mean number of remedies cited by gender and age categories.

The mean number of remedies quoted by interviewees is reported by age group and gender (Figure 7). A generation gap in knowledge regarding folk remedies for SSTI's was evident. There is a statistically significant difference (p < 0.05) in knowledge of folk remedies for the skin between the youngest subset of women sampled (21–35 years old) and women 36 years and older. This may be explained by recent shifts in the socioeconomic patterns of the region. Prior to the opening of the Fiat automobile factory and all of the associated parts factories in the region, women seldom participated in the workforce. When the factories opened nearly 15 years ago, a large shift in women's socioeconomic placement occurred. Young women entered the workforce and no longer regularly participated in outdoor activities, such as upkeep of the family gardens and vineyards, thus creating a significant gap between them and nature. It also fractured the daily interaction with the older women, who are the primary keepers of TK and responsible for the oral passage to younger generations. This socioeconomic shift, undoubtedly, played a significant role in the decline of passing TK on to this youngest generation of women.

There was no significant difference between men in different age groups and men and women were roughly equivalent in oldest subsets sampled (51–70 and 71+ years old). This lack of difference in TK for men may be explained by historic emigration patterns. A large portion of men from the oldest subsets sampled emigrated to northern Italy and Switzerland in their youth for work. Later in life, these men returned to the community of their birth, but the substantial number of years away from their native environment and traditions was detrimental to their level of TK. In addition, younger men, like the young women, did not acquire much TK due to dynamic of the local economy.

Another pattern in TK levels related to the size of communities was notable. Younger men from the smaller communities (primarily the localities), who were actively involved in agriculture – either in farming crops or pastoral activities, had much higher levels of TK for skin remedies than young men from larger, less isolated communities. For example, one young shepherd from the locality of Sant'Andrea was able to describe more than 40 remedies related to the skin. Young men who either worked in factory jobs or were unemployed from a larger community, like Ripacandida, could typically only cite 4 to 7 remedies. This reflects the importance of daily interaction with the environment and its role in the acquisition and retention of TK of plant derived remedies. A qualitative analysis of the data also reflects a greater level of TK retention and transmission between generations in the smallest communities.

In summary, the likelihood of a continued decline in TK transmission across generations is great and will be most notable amongst women. Socioeconomic factors and shifts in the dynamics of women's roles in the home have perhaps the greatest influence on this paradigm. Stronger dependence on allopathic, rather than traditional medical care is expected in the coming years.

## Conclusion

We identified 165 remedies derived from plant, animal, mineral and industrial sources with particular relevance to skin and soft tissue infection. Of the remedies reported, 110 have never before been published in the mainland southern Italian ethnomedical literature. Moreover, some of the botanical sources for SSTI remedies have never been investigated for their antimicrobial properties and further investigation of the phytochemical constituents of these species is recommended.

Transfer of knowledge regarding household medicine is declining, especially amongst women. These findings are important because women have traditionally been the keepers of knowledge regarding folk remedies and the providers of household medicine. This loss of knowledge is intrinsically linked with shifting socioeconomic dynamics in the area and is not expected to be reversed. This reinforces the importance of recording ethnomedical and ethnobiological data now before it is lost with the passing of the oldest generations.

## Competing interests

The author(s) declare that they have no competing interests.

## Authors' contributions

CQ carried out the field research, analyzed the data and wrote the manuscript. BB and AP participated in the design of the study, taxonomic identification of botanic species, and helped to revise the manuscript. All authors read and approved the final manuscript.

## Supplementary Material

Additional File 1 Popular uses of botanical materials for dermatological conditions and topical (external) applications. Click here for file

Additional File 2 Popular uses of animal, mineral, or industrial materials for dermatological conditions and topical (external) applications. Click here for file
