# Supplementary material for: Dermatological remedies in the traditional pharmacopoeia of Vulture-Alto Bradano, inland southern Italy
Source: J Ethnobiol Ethnomed. 2008 Feb 6;4:5. doi: 10.1186/1746-4269-4-5 (PMC2275234; doi:10.1186/1746-4269-4-5)
Supplement: Additional File 1 — Popular uses of botanical materials for dermatological conditions and topical (external) applications. [file 1746-4269-4-5-S1.PDF]

Additional file 1. Popular uses of botanical materials for dermatological conditions and topical (external) applications

Botanical taxon (voucher specimen code)	*Botanical family*	*Vernacular name in Vulture-Alto Bradano*	*English name*	*Status*	*Part(s) used*	*Preparation and application*	*Popular use*	*Consensus index* ♦<10% ♦♦ 10-20% ♦♦♦ 21-30% ♦♦♦♦ 31-40% ♦♦♦♦♦ >40%	*Records of similar use in other Italian ethnobotanical studies conducted in mainland southern Italy*
Aesculus hippocastanum L.	Sapindaceae	*castagna bastarda*	horse chestnut	W	fruit	Fresh fruits are split in half and seeped in alcohol for 40 days and then used to massage swollen or sore legs.	Analgesic; anti-oedemic	♦	None
					bark and leaves	A decoction is made of the bark and leaves then mixed with olive oil or pig fat to make an ointment. It is applied topically.	Anti-haemorrhoid	♦	None
Allium cepa L. (CQ-206)	Alliaceae	*cipudda*	onion	C	bulb	Slice of bulb is warmed, and then a drop of olive oil and sugar is added. This is then topically applied.	Anti-furuncle; anti-abscess	♦	[1, 2]
					bulb	Slice of fresh bulb is rubbed on burned skin.	Vulnerary (for burn wounds)	♦	None
					bulb membrane	Applied topically (like a Band-Aid) to small lacerations.	Haemostatic	♦♦	[3]
					leaves	Fresh leaves are used to tie strips of bark from *Ulmus*	Splint for broken or	♦	None

						minor L. around the affected limb.	severely bruised bones		
Allium sativum L.	Alliaceae	*agljē*	garlic	C	bulb	Piece of bulb is crushed and rubbed onto nipple of breast-feeding mother.	Wean baby from breast milk	♦	None
						Bulb is boiled, and then while still hot, it is rubbed onto a callus.	Anti-callus; analgesic (for callus)	♦	[4, 5]
						Pieces of fresh bulb (small cloves) are kept in mouth to suck on.	Against sore throat	♦	None
						Fresh pieces of bulb are chewed.	Against toothache; anti-gingivitis	♦	[5, 6]
						Fresh bulb is rubbed onto the skin where stung by an insect.	Anti-sting/anti-itch (for insect bite)	♦♦	[1, 2, 4, 7]
Arum italicum Mill. (CQ-175)	Araceae	*frugnē n cuttē*	Italian lords and ladies	W	leaves	Fresh leaf applied topically. It will "stick" to the skin.	Analgesic (for insect stings and burns); vulnerary (for burn wounds)	♦♦	None
Arundo donax L. (CQ-146)	Poaceae	*cannē*	giant reed	W	white hemi-cellulose membrane at node	Topically applied to lacerations.	Haemostatic	♦♦♦♦	[1-3, 5]
						Topically applied to mouth sores on lips (but	Anti-herpes (of the	♦	None

						not inside the mouth or on tongue).	mouth)		
					stem	Stem is cut so that it will fit onto the fingers. These *canned* are worn to protect the fingers during the grain harvest.	Physical protection of fingers	♦♦	None
						Green stem is cut into 9 tooth-size pieces which are used in the ritual healing of toothache.	Against toothache	♦	None
Ballota nigra L. (CQ-160)	Lamiaceae	*erba cane*	black horehound	W	aerial parts	Decoction is used as a skin wash.	Anti-inflammatory<br>Haemostatic	♦<br>♦	None<br>[2]
Brassica oleracea L.	Brassicaceae	*cavolo*	cabbage	C	leaf	Heated and topically applied for pain relief.	Anti-rheumatic; anti-arthritis; analgesic	♦	[5, 7]
						Fresh leaf is wrapped around injured area of limb, and then held in place with a bandage. Frequently used on horse legs.	♣ Analgesic and anti-inflammatory for tendonitis	♦	None
						Fresh leaf applied to engorged breast of lactating woman.	anti-lactic; anti-mastitis	♦	[2]
Cannabis sativa L.	Cannabaceae	*canapē*	hemp	W	fibers	Hemp fibers (*stoppē*) are coated in well-beaten egg white and used to make a hard cast (*stoppata*) for setting bones.	Cast for broken or severely bruised bones	♦	None

Capsicum annuum L.	Solanaceae	*pepperoni picantē*	sweet pepper	C	fruit	Dried and rubbed onto nipple of breast-feeding mother.	Wean baby from breast milk	♦	None
Cyclamen hederifolium L. (CQ-186)	Myrsinaceae	*ciclamino*	hardy cyclamen	W	tuber	Black skin of fresh tuber is removed, and then the rest of tuber is grated to make a poultice for topical applications.	Anti-wart	♦	None
Ecballium elaterium (L.) A. Richard (CQ-169)	Cucurbitaceae	*u cuppē ti ridzē*	squirting cucumber	W	leaf	Heated and topically applied.	Anti-abscess	♦	None
						Fresh leaf is wrapped around painful limb.	Anti-rheumatic; anti-inflammatory; analgesic	♦	None
Ficus carica L. (CQ-173)	Moraceae	*fēchē*	fig	C	latex from immature fruit	Topically applied.	Anti-callus; anti-corn (feet)	♦	[7]
							Anti-wart	♦♦	[1, 2, 5-8]
							Anti-itch (for itchy dermatitis); anti-inflammatory	♦	None
							Anti-sting/anti-itch (for insect bites)	♦	[2, 4, 7]
						Topically applied to	Anti-herpes	♦	None

						mouth sores on lips (but not inside the mouth or on tongue).	(of the mouth)		
					leaf	Fresh leaf is placed on breast as compress.	Anti-mastitis	♦	None
Foeniculum vulgare ssp. *vulgare* Mill. (CQ-196)	Apiaceae	*fēnocchē*	fennel	W	seeds	Seeds are chewed by another person and the resulting aromatic breath is blown into the inflamed or red eyes of the patient.	Anti-conjunctivitis	♦	None
Hordeum vulgare L.	Poaceae	*orzo*	barley	C	fruits (grains)	Boiled and placed in cloth to be used as warm compress that is applied to the throat.	Against sore throat	♦	None
Hypericum perforatum L. (CQ-183)	Hypericaceae	*erva pē rē cuttē; iperico*	St. John's Wort	W	aerial parts	Fresh aerial parts are used to make an oleolite (heated in olive oil). When the oil has cooled, it is used to massage injured or swollen horse legs.	♣ Anti-inflammatory ; emollient; vulnerary	♦	[4]
Juglans regia L. (CQ-181)	Juglandaceae	*noce*	walnut	SC	bark	Decoction used as mouth wash or gargle.	Against toothache	♦	None
					flowers	Topically applied as ritual object in spiritual healing of *mal d'arco* (rainbow illness).	Against "Rainbow illness"	♦	None
					leaves	Fresh leaves topically applied to areas of swelling from varicose veins. This is done at	Heals varicose veins; reduces swelling of	♦	[6]

						night and the leaves are held in place with a bandage.	legs		
Malva sylvestris L. (CQ-156)	Malvaceae	*malvē*	common mallow	W	aerial parts (or just stems)	Decoction – once cooled, it is used as a wash for infants. Some also make the decoction together with the dried aerial parts of *Matricaria recutita.*	Anti-dermatitis (babies); Against heat- and diaper-rash in babies	♦	None
						Decoction is used as a mouth wash.	Against toothache (dental abscess)	♦	[4, 7, 9]
					leaves	Fresh leaf applied topically; changed 2-3 times a day.	Anti-bruise	♦	None
						Fresh leaf is heated over a fire and applied topically as compress; compress is changed 2-3 times a day.	Anti-furuncle; anti-abscess	♦♦	[5, 8]
						Fresh leaves are boiled and placed into cloth sack to use as a warm compress; compress is changed 2-3 times a day.	Anti-mastitis	♦♦	[5]
						Leaves are boiled and placed into a cloth sack to use as a compress that is held on the jaw or cheek.	Against toothache (dental abscess)	♦	None
						Leaves are boiled and packed into the space where rotten teeth have	Against toothache (dental	♦	None

						fallen out or areas of acute pain from toothache.	abscess)		
Marrubium vulgare L. (CQ-170)	Lamiaceae	*marruggē*	white horehound	W	aerial parts	Decoction is made and used as a wash for the affected area. The decoction is left on the skin to air dry. This is repeated 2-3 times a day until healed.	♣ Anti-furuncle; anti-abscess; anti-cyst (used on humans and livestock)	♦♦♦	[2, 9]
							Anti-dermatitis	♦	[5]
							Anti-fungal (for foot fungus)	♦	None
							Anti-haemorrhoid	♦	[9]
							Anti-wart	♦	None
							Panacea	♦♦♦♦♦	[1, 3, 5, 9]
Matricaria recutita L. (CQ-118)	Asteraceae	*camomilla*	German chamomile	W	stems	Dried, then made into decoction that is used as an eye wash. Eyes are washed 3 times a day until healed. A cloth compress soaked in this decoction water can also be applied to the eyes.	Anti-conjunctivitis; anti-inflammatory (of eye)	♦♦♦	[1, 2, 8]
					aerial parts (or just stems)	Dried, then made into decoction that is used as a facial wash.	Face cleansing; anti-acne	♦	[10]

						Dried, then made into decoction that is used to wash or bathe babies with heat- or diaper-rash. Some also make the decoction together with the dried aerial parts of *Malva sylvestris* L.	Anti-dermatitis; anti-rash; anti-inflammatory	♦	None
						Dried, then made into decoction for washing hair.	Wash for oily hair	♦	None
							Hair lightener	♦	None
Olea europaea L. (CQ-197)	Oleaceae	*aulivē*	olive	C	leaves	Decoction with rosemary leaves (*Rosmarinus officinalis* L.) is used as a foot bath.	Relieve foot swelling	♦	None
					oil	Topically applied to burn wound. This must be done in secret immediately after burn injury occurs.	Vulnerary and emoillient (for burn wounds)	♦♦	[5-7]
						Rubbed onto the skin several times a day to treat the folk illness *Fuoco di Sant'Antonio* (St. Anthony's Fire), or shingles.	Anti-shingles; anti-inflammatory	♦	None
						Rubbed onto the skin of babies with diaper- or heat -rash.	Anti-dermatitis; anti-inflammatory	♦	None
						Rubbed onto the crown of	Against	♦	None

						baby's head, then hair is brushed with soft brush or comb.	cradle-cap (in infants)		
						Warm oil is applied to animal hooves.	♣ Vulnerary for cracked hooves or chapped skin near hooves	♦	None
						Topical application to diaper area on infants.	Against diaper rash	♦	None
						Topical applications to dry skin.	Emollient	♦♦	[6]
						Rubbed into the hair and scalp in ritual treatment for scabies and lice.	Anti-scabies; anti-lice	♦	[8]
Origanum heracleoticum L. (CQ-207)	Lamiaceae	*rigano*	oregano	W	leaves	Dried leaves are mixed with olive oil and made into a compress that is applied to jaw.	Against toothache	♦	[1]
Oryza sativa L.	Poaceae	*riso*	rice	C	grains	Rice grains (store bought) are boiled until over-cooked, then applied topically.	Suppurative; anti-furuncle; anti-abscess	♦	None
Parietaria diffusa Mert. & Koch (CQ-212)	Urticaceae	*erba vjntē*	pellitory of the wall	W	aerial parts	A decoction of aerial parts is used as a mouth wash.	Against toothache; anti-gingivitis	♦	None
Plantago major L. (CQ-225)	Plantaginaceae	*cintu nirvi*	common plantain	W	leaves	Fresh leaves are crushed with a stone then applied topically.	Anti-rheumatic; anti-arthritic; analgesic; anti-	♦♦	None

							inflammatory		
							anti-abscess; anti-boil; suppurative	♦♦♦♦	[1, 4, 8, 9]
							Vulnerary (for burns)	♦♦♦	None
Rosmarinus officinalis L. (CQ-113)	Lamiaceae	*rosamarē nē*	rosemary	C, W	leaves	Decoction with olive leaves (*Olea europaea* L.) is used as a foot bath.	Relieve foot swelling	♦	None
Rubus ulmifolius Schott (CQ-164)	Rosaceae	*mora*	elmleaf blackberry	W	leaves	A layer of aged pig fat (*la sugnē fracidē*) is placed on affected area of skin, and then fresh leaves are placed on top of the fat. This is wrapped with a bandage to hold the leaf and fat in place. (Some also cite the application of the leaf directly to furuncle and abscess without using pig fat.)	Anti-furuncle; vulnerary (for lacerations); anti-abscess; anti-carbuncle; suppurative	♦♦	[4, 7]
						Fresh leaves are applied topically to skin to treat dog bite. The leaves are held in place with a bandage.	Vulnerary and anti-infective (for dog-bite)	♦	None
					root	A decoction of the fresh root is made for use as a hair wash.	Prevent/stop hair loss	♦	None
Rumex crispus L. (CQ-171)	Polygonaceae	*patsa*	curled dock	W	leaves	Leaves are warmed over a fire; the cortex is removed and placed in olive oil.	Vulnerary (for burn wounds)	♦	None

						This is topically applied to burn wounds.			
Ruta graveolens L.	Rutaceae	*ruta*	rue	W	aerial parts	Dried then made into a decoction that is used as an external wash for humans and animals.	♣ anti-furuncle; anti-abscess; anti-acne	♦	None
						Decoction of aerial parts is drunk or used as an external wash.	Panacea	♦	None
Sambucus ebulus L. (CQ-180)	Adoxaceae	*Iervolē*	dwarf elder	W	flowers	When flowers turn black, they are placed in shoes and walked in (without socks) for 3 days.	Prevents formation of blisters and foot sores	♦	None
					fruit	Juice from mature fruits is applied to open wounds in animals to detract flies and other insects from the wound.	♣ Fly and insect repellent for open wounds	♦	[11]
					leaves	Fresh leaf is wrapped around foot before putting on shoes.	Prevents formation of blisters; sweat absorbent; "toughens" foot skin	♦	[2]
Sambucus nigra L. (CQ-151)	Adoxaceae	*savēuchē*	elder	W	flowers	Dried then made into infusion for use as a foot bath.	Vulnerary for foot sores; emollient for dry, cracked feet	♦	[8, 10]
					leaves	Fresh leaves topically applied.	Anti-rheumatic;	♦	[3, 6]

							analgesic; Anti-inflammatory		
							Anti-sting/anti-itch (for insect bites)	♦	[1, 5]
							Haemostatic (for lacerations)	♦	None
							Anti-boil; anti-abscess; suppurative	♦	[7]
Solanum lycopersicum L.	Solanaceae	*pomodoro*	tomato	C	fruit	Fruit pulp is rubbed onto the skin.	Heals insect stings (from wasps and bees)	♦	None
							Anti-callus	♦	None
Solanum tuberosum L.	Solanaceae	*potata*	potato	C	tuber	Fresh slice of tuber is topically applied.	Vulnerary (for burn wounds)	♦♦	[3, 5, 7, 10]
						Fresh slice of tuber is topically applied.	Anti-bruise	♦	None
						Fresh slice of tuber is grated and applied to eyes.	Anti-inflammatory (for eyes); eye moisturizer	♦	[3, 5, 12]
						Fresh skin of tuber is peeled off and applied as a compress on the jaw or	Analgesic (against tooth-ache)	♦	None

						cheek over area of toothache pain.			
						Fresh slice of tuber is placed on the forehead until headache is relieved.	Anti-headache	♦	[5]
Tussilago farfara L. (CQ-202)	Asteraceae	*stampē kavalē1*	coltsfoot	W	leaves	Fresh leaf topically applied to affected area.	Anti-furuncle; suppurative	♦	[3, 7]
							Vulnerary (for burn wounds)	♦	None
						Fresh leaves applied as ritual objects in healing ceremony for *pesta cane* (dog paw print illness) in which the leaves are laid in a cross over the lower back of infants that won't grow.	Against *pesta cane* (dog paw print illness)	♦	None
Ulmus minor L. (CQ-195)	Ulmaceae	*olmo*	elm	W	bark	Strips of bark are used to make a splint and the bark is tied around the affected limb with the leaves of *Allium cepa* L.	Splint for broken or severely bruised bones	♦	None
Umbilicus rupestris (Salisb.) Dandy (CQ-157)	Crassulaceae	*favē alla mersē; grassossē; cuppētieddē ; grassognē*	navelwort	W	leaves	Fresh leaf is topically applied to affected area. Most cite the extraction, or "peeling off" of the leaf "skin" (epithelium) before using.	Anti-corn (feet); anti-callus (feet)	♦♦	[7]
							Heal ingrown toenails	♦♦	None

							Anti-furuncle; anti-abscess; suppurative	♦	[1-3, 13]
							Relieves foot swelling; anti-inflammatory	♦	[2]
							Vulnerary (also for lacerations)	♦	None
						Fresh leaf is heated and applied to jaw or cheek.	Against toothache	♦	None
Urtica dioica L. (CQ-179)	Urticaceae	*ardiga*	common nettle	W	leaves	Fresh leaves are topically applied.	Anti-rheumatic; anti-arthritis	♦	[4, 9, 10]
						Decoction used as hair wash.	Wash for oily hair; strengthens weak hair	♦	[8, 10]
Verbascum sinuatum L. (CQ-218)	Scrophularia-ceae	*varvaschē*	wavyleaf mullein	W	leaves	Decoction in red wine (*Vitis vinifera* var. *aglianico*) is used as a mouth wash and gargle. It isn't swallowed because it is considered poisonous.	Analgesic (for toothache and mouth sores)	♦	None
Vitis vinifera var. *aglianico* (CQ-209)	Vitaceae	*vētē*	grape vine	C	vinegar	Cloth is bathed in vinegar and placed on forehead.	Febrifuge	♦	None
						Hair is washed first with soap and water, then with vinegar.	Wash for oily hair	♦	[3]
						Mixed with salt and	Against skin	♦	None

		rubbed on skin.	nodules; anti-cyst		
		Mixed with salt and used as mouth wash.	Against toothache; anti-gingivitis	♦	None
		Mixed with *verderame* (oxidized copper, a fungicide used on cultivated plants) and used to treat *canero del fatone,* or cracked/ wounded horse hooves.	♣ Heals cracked or wounded horse hooves	♦	None
	wine	Used to wash wounds and lacerations (for humans and animals).	♣ Anti-septic	♦♦♦♦	[14]
		Decoction with the leaves of *Verbascum sinuatum* L. is used as a mouth wash and gargle. It isn't swallowed because it is considered poisonous.	Analgesic (for toothache and mouth sores)	♦	None
		Mixed with bread crumbs, and then rubbed onto skin to treat contact dermatitis caused by a stinging caterpillar crawling on skin.	Anti-dermatitis; anti-inflammatory	♦	None
		Applied topically in ritual healing of *mal vjntē* (wind illness).	Against *mal vjntē* (wind illness)	♦	None
	wine or vinegar	Mixed with durum wheat flour, and then used to	Cast for broken or	♦	None

		coat a rough canvas material (*stoppē*) to make a hard cast for setting bones.	severely bruised bones

Status: C: cultivated; SC: semi-cultivated (including plants managed in the wild); W: wild. *Consensus Index/ Spontaneous Quotation Frequency for remedies:*

♦: quoted by < 10% of informants; ♦♦ quoted by 10- 20% of informants; ♦♦♦: quoted by 21-30% of informants; ♦♦♦♦: quoted by 31-40% of informants; ♦♦♦♦♦: quoted by > 41% of informants. ♣: Ethnoveterinary application.

References

1. Pieroni A, Quave CL: **Traditional pharmacopoeias and medicines among Albanians and Italians in southern Italy: A comparison.** *Journal of Ethnopharmacology* 2005, **101:**258-270.

2. Pieroni A, Quave CL, Santoro RF: **Folk pharmaceutical knowledge in the territory of the Dolomiti Lucane, inland southern Italy.** *Journal of Ethnopharmacology* 2004, **95:**373-384.

3. Pieroni A, Quave C, Nebel S, Heinrich M: **Ethnopharmacy of the ethnic Albanians (Arbëreshë) of northern Basilicata, Italy.** *Fitoterapia* 2002, **73:**217-241.

4. Scherrer AM, Motti R, Weckerle CS: **Traditional plant use in the areas of Monte Vesole and Ascea, Cilento National Park (Campania, Southern Italy).** *Journal of Ethnopharmacology* 2005, **97:**129-143.

5. Passalacqua NG, Guarrera PM, De Fine G: **Contribution to the knowledge of the folk plant medicine in Calabria region (Southern Italy).** *Fitoterapia* 2007, **78:**52-68.

6. De Feo V, Aquino R, Menghini A, Ramundo E, Senatore F: **Traditional phytotherapy in the Peninsula Sorrentina, Campania, southern Italy.** *Journal of Ethnopharmacology* 1992, **36:**113-125.

7. Guarrera PM: **Traditional phyotherapy in Central Italy (Marche, Abruzzo, and Latium).** *Fitoterapia* 2005, **76:**1-25.

8. Guarrera PM, Salerno G, Caneva G: **Folk phytotherapeutical plants from Maratea area (Basilicata, Italy).** *Journal of Ethnopharmacology* 2005, **99:**367-378.

9. Guarrera P, Leporatti L: **Ethnobotanical remarks on Central and Southern Italy.** *Journal of Ethnobiology and Ethnomedicine* 2007, **3:**23.

10. De Feo V, Senatore F: **Medicinal plants and phytotherapy in the Amalfitan Coast, Salerno Province, Campania, southern Italy.** *Journal of Ethnopharmacology* 1993, **39:**39-51.

11. Guarrera PM: **Traditional antihelmintic, antiparasitic and repellent uses of plants in Central Italy.** *Journal of Ethnopharmacology* 1999, **68:**183-192.

12. Guarrera PM: **Food medicine and minor nourishment in the folk traditions of Central Italy (Marche, Abruzzo and Latium).** *Fitoterapia* 2003, **74:**515-544.

13. Palmese MT, Uncini Manganelli RE, Tomei PE: **An ethno-pharmacobotanical survey in the Sarrabus district (south-east Sardinia).** *Fitoterapia* 2001, **72:**619-643.

14. Pieroni A, Howard P, Volpato G, Santoro RF: **Natural remedies and nutraceuticals used in ethnoveterinary practices in inland southern Italy.** *Veterinary Research Communications* 2004, **28:**55-80.
