# Supplementary material for: Dermatological remedies in the traditional pharmacopoeia of Vulture-Alto Bradano, inland southern Italy
Source: J Ethnobiol Ethnomed. 2008 Feb 6;4:5. doi: 10.1186/1746-4269-4-5 (PMC2275234; doi:10.1186/1746-4269-4-5)
Supplement: Additional File 2 — Popular uses of animal, mineral, or industrial materials for dermatological conditions and topical (external) applications. [file 1746-4269-4-5-S2.PDF]

Additional file 2. Popular uses of animal, mineral, or industrial materials for dermatological conditions and topical (external) applications

Ingredient	*Vernacular name in Vulture-Alto Bradano*	*Preparation and application*	*Popular Use*	*Consensus index ♦<10% ♦♦ 10-20% ♦♦♦ 21-30% ♦♦♦♦ 31-40% ♦♦♦♦♦ >40%*	*Records of similar use in other Italian ethnobotanical studies conducted in mainland southern Italy*
Ash	*cinerē*	Ashes from fireplace are added to a pail of water and mixed. This is used for washing hair, dishes, and clothing.	Hair wash	♦	None
Breastfeeding	*lattē alla reversē*	Nursing mothers with mastitis must breast-feed their infants using the "reverse" or "football" position.	Anti-mastitis	♦♦	None
Brick or Tile	*mattone caldo*	A brick or clay tile is heated, and then applied to the skin over the affected area.	Heals bruises; anti-mastitis	♦	None
	mattone fredo	A cold tile is held on the abdomen for gastritis.	Anti-gastritis	♦	None
Cigarette filter	*cica della sigaretta*	Filter of cigarette (tobacco is removed) is placed inside mouth and applied directly to the area of toothache pain.	Analgesic (for toothache)	♦	None
Cooked cream (from cow's milk)	*panna cottē*	Cooked cream is applied topically.	Anti-furuncle	♦	[1]
Copper sulphate (chemical fungicide for cultivated plants)	*verderammē*	Mixed with vinegar from *Vitis vinifera* var. *aglianico* and used to treat *canero del fatone,* or cracked/ wounded horse hooves.	♣ Heals cracked or wounded animal hooves and the chapped skin around the hooves	♦	None

		Mixed with water and sometimes salt. It is used to soak injured or swollen feet. This is rarely used on humans because it produces a "burning" sensation of the skin.	Heals chapped skin and swelling of feet	♦♦	None
			♣ Heals cracked or wounded animal hooves and the chapped skin around the hooves	♦♦	[2]
		Ground into a powder form that is sprinkled onto animal hooves.	♣ Heals cracked or wounded animal hooves and the chapped skin around the hooves	♦	[1]
Cow's milk	*u latte freschē*	Fresh milk is used to wash facial skin or dry skin.	Anti-wrinkle; emollient	♦	None
Dog hair	*pelo di cane*	Take a hair from the dog that bit you and apply topically to bite wound.	Heal dog bite	♦	None
Egg white	*bianco di uova*	Egg white is beaten well, and then used to coat hemp (*Cannabis sativa* L.) fiber (*stoppē*) to make a hard cast (*stoppata*) for setting bones.	Cast for broken or severely bruised bones	♦	None
Egg Yolk	*rosso di uovo*	Egg yolk is mixed with the yeast from fresh pasta dough and is then applied topically.	Anti-furuncle; anti-abscess	♦	None
Hedgehog bile	*bile dē riccē*	Bile of a freshly killed hedgehog is fed to babies that won't stop crying	Calms crying babies	♦	None
Honey	*miele*	Topically applied to the nipple between breast-feeding sessions.	Emollient for chapped nipples (of breast-feeding mothers)	♦	None

Human breast milk	*lattē dē mama*	Fresh milk is expressed into the infected eyes of babies (also sometimes for adults). Some state the milk must be from a mother who nurses a son. Others say the milk must be from a mother nursing her first born child. Less commonly, fresh milk from an ass or goat is used.	Anti-conjunctivitis	♦♦♦	[1]
		Breast milk is expressed and rubbed onto the nipple.	Heals chapped skin of nipple (for breast-feeding women)	♦	None
		Breast milk is expressed and rubbed onto an infant's scalp. This is followed by brushing the hair of the infant to remove the excess skin.	Against cradle-cap	♦	None
Human urine	*piscē*	Used to wash and clean lacerations.	Haemostatic; antiseptic	♦♦	[1]
		First urine of the day is used to wash eyes	Anti-conjunctivitis	♦	None
Knife (must have a black handle)	*coltello con u manicho nero*	The knife is used to make the mark of a cross (indentations – not cuts) onto the skin over the area of an insect sting. The metal of this knife will "absorb the poison" and take away the sting.	Heals insect stings; analgesic	♦	None
Matches (sulfur based)	*fiammifirē dē zolfē*	Match is lit and held close to a laceration.	Haemostatic	♦	None
		Match is lit and used to burn warts.	Anti-wart	♦	None
Pasta dough (that is stolen)	*pasta rubattē*	Pasta dough is stolen in secret, and then rubbed on the skin. The pasta dough must then be hidden or thrown away in secret. When the dough "dries up", so will the wart.	Anti-wart	♦	None

Pasta dough (homemade)	*pasta fattē in casē*	Pasta dough is rolled out into a thin strip that is applied topically to areas of muscle pain.	Analgesic (for muscle pains or injured joints)	♦	None
Pork fat (from dorsal back fat – it is aged and kept in cantina hanging on a hook)	*sugna fracidē*	Applied topically to sore chapped nipples (for breast-feeding women) or to chapped skin in general.	Emollient	♦	None
		A thin layer of fat is placed on affected area of skin, and then fresh leaves of *Rubus ulmifolius* Schott are placed on top of the fat.	Anti-furuncle; vulnerary (for lacerations); anti-abscess; suppurative	♦♦	[3]
		Layer of fat is applied to the chest. This is then covered with a warm towel.	Anti-bronchitis	♦	None
		Fat is rubbed onto animal wounds and lacerations to protect the injured area and promote healing.	♣ Vulnerary	♦	[2]
		Layer of fat is applied over the burn wound. Or, the fat may first be boiled, and then placed on burn wound.	Vulnerary (for burn wounds)	♦	None
		Applied topically to the udders of milk-producing animals, especially cows.	♣ Emollient for chapped udders; protects against flies	♦	None
Pork lard	*lardo di maiale*	Lard is boiled, and then when cooled it is applied topically to burn wounds.	Vulnerary (for burn wounds)	♦	None
Pumice stone powder	*pietra pomice*	Powder from pumice stone is topically applied to wounds.	Vulnerary	♦	None
Rock (unidentified)	*pietra*	Rock is rubbed onto burn wounds.	Vulnerary (for burn wounds)	♦	None

Salt (that is stolen)	*sale rubbattē*	A handful of salt is stolen in secret, and then rubbed on the skin. The salt must then be hidden or thrown away in secret.	Anti-wart	♦	None
Salt and water	*bagnē a sale e acqua*	A warm bath of salt and water is used to soak feet.	Heals swollen, sore feet	♦♦	None
Slug (*Arion hortensis* Férussac)	*marruculē; lummachē senza guscio*	Live slug is swallowed whole.	Anti-ulcer (stomach ulcer); anti-gastritis	♦♦♦	None
		Mucous from live slug is rubbed onto skin, then animal is hung out in sunshine – when it dries up, the wart will also "dry up".	Anti-wart	♦♦♦	[1]
		Mucous from live slug is rubbed onto skin.	Vulnerary; against dermatitis; anti-inflammatory	♦♦	None
			Anti-callus	♦	None
		Mucous from live slug is rubbed onto skin for facial cleansing.	Anti-acne; emollient	♦	None
Soot from fireplace	*fuliggine*	Black soot is topically applied to wounds.	Vulnerary	♦	None
Tractor oil (old or burnt oil)	*olio bruggiatē*	Old tractor oil is mixed with ashes from burnt hay to make a poultice which is applied topically to lacerations and wounds in animals (especially for horses and mules).	♣ Cicatrizing	♦	None
		Old tractor oil is brushed onto animal's (usually for horses, mules, and donkeys) coat to detach blood-sucking insects known as *tafani* from the animal's skin.	♣ Insecticide	♦	None

Water	*acqua bollente*	For fingertip-pricks (such as with a needle), the affected fingertip is quickly dipped into boiling water three times.	Against pains from fingertip-prick	♦	None
Water foam	*schiumma d'acqua*	Foam from water coming from natural spring fountains is topically applied to warts.	Anti-wart	♦	None
Wood affected by woodworms (*Anobium punctatum*)	*leuna imbracētatē*	Powder from this wood is rubbed onto the top of an infant's head.	Against cradle-cap	♦	None
		Powder from this wood is topically applied to diaper area in babies.	Against diaper- or heat-rash	♦	[1]

Consensus Index/ Spontaneous Quotation Frequency for remedies: ♦: quoted by < 10% of informants; ♦♦ quoted by 10-20% of informants; ♦♦♦: quoted by 21-30% of informants; ♦♦♦♦: quoted by 31-40% of informants; ♦♦♦♦♦: quoted by > 40% of informants. ♣: Ethnoveterinary application.

References

1. Pieroni A, Quave CL, Santoro RF: **Folk pharmaceutical knowledge in the territory of the Dolomiti Lucane, inland southern Italy.** *Journal of Ethnopharmacology* 2004, **95:**373-384.

2. Pieroni A, Howard P, Volpato G, Santoro RF: **Natural remedies and nutraceuticals used in ethnoveterinary practices in inland southern Italy.** *Veterinary Research Communications* 2004, **28:**55-80.

3. Scherrer AM, Motti R, Weckerle CS: **Traditional plant use in the areas of Monte Vesole and Ascea, Cilento National Park (Campania, Southern Italy).** *Journal of Ethnopharmacology* 2005, **97:**129-143.
